# Overexpression of a modified 6-phosphofructo-1-kinase results in an increased itaconic acid productivity in *Aspergillus niger*

**DOI:** 10.1186/2191-0855-3-57

**Published:** 2013-09-13

**Authors:** Laura van der Straat, Juan A Tamayo-Ramos, Tom Schonewille, Leo H de Graaff

**Affiliations:** 1 Microbial Systems Biology, Laboratory of Systems and Synthetic Biology, Wageningen University, Dreijenplein 10, 6703, HB, Wageningen, Netherlands

**Keywords:** Itaconic acid, *Aspergillus niger*, Modified 6-phosphofructo-1-kinase, *cis*-aconitate decarboxylase *cadA*, Mitochondrial transporter *mttA*, Plasmamembrane transporter *mfsA*

## Abstract

A modified 6-phosphofructo-1-kinase was expressed in a citrate producing *Aspergillus niger* strain in combination with *cis*-aconitate decarboxylase from *Aspergillus terreus* to study the effect on the production of itaconic acid. The modified *pfkA* gene was also expressed in combination with the itaconic acid biosynthetic cluster from *A. terreus*, which consists of *cis-*aconitate decarboxylase *cadA*, a putative mitochondrial transporter *mttA* and a putative plasmamembrane transporter *mfsA*. The combined expression of *pfkA* and *cadA* resulted in increased citrate levels, but did not show increased itaconic acid levels. The combined expression of *pfkA* with the itaconic acid biosynthetic cluster resulted in significantly increased itaconic acid production at earlier time points. Also the itaconic acid productivity increased significantly. The maximum itaconic acid productivity that was reached under these conditions was 0.15 g/L/h, which is only a factor 17 lower than the 2.5 g/L/h that according to the US Department of Energy should be achieved to have an economically feasible production process.

## Introduction

The bio-based production of chemicals from second-generation plant biomass is both economically and scientifically challenging. The cost-efficient production of chemicals requires highly efficient production processes based on highly optimized production organisms. One of the promising production organisms in this respect is *Aspergillus niger.* This filamentous fungus is industrially used for the production of plant cell wall hydrolyzing enzymes and for the production of metabolites like citrate. Based on its current use, *A. niger* can relatively easily be modified for the production of bio-based chemicals. Itaconic acid is a metabolic derivative of citric acid and therefore can potentially be produced in *A. niger.* Itaconic acid (methyl succinic acid) is a C5 dicarboxylic acid. It is one of the top twelve building block chemicals that can be produced from plant biomass sugars *via* a fermentative process (Werpy and Petersen 2004). The methylene group of itaconic acid can participate in polymerization reactions and on the basis of this characteristic itaconic acid can be used for the production of synthetic polymers (Kubicek et al. 2010). It is further used as a bioactive compound in agriculture, pharmacy and as a medicine (Okabe et al. 2009). Itaconic acid can also be used as a starting compound for enzymatic transformations to form useful poly-functional building blocks (Ferraboschi et al. 1994).

Nowadays, *Aspergillus terreus* is mostly used for the commercial production of itaconic acid in a submerged fermentation process (Okabe et al. 2009; Willke and Vorlop 2001). The metabolic pathway for the production of itaconic acid is similar to the metabolic pathway of citric acid production in *A. niger*. Given this similarity in biosynthesis in *A. niger* we have started to develop an itaconic acid production process based on *A. niger* citric acid producing strains.

Citric acid is commercially produced using *A. niger* reaching production levels of more than 200 g/L, while *A. terreus* reaches itaconic acid production levels of 80 g/L (Kuenz et al. 2012). Therefore *A. niger* is the host of choice for our research. *A. niger* is not able to produce itaconic acid naturally since it lacks the essential gene *cadA* encoding *cis*-aconitate decarboxylase*.* The *cadA* gene was identified *via* a clone-based transcriptomics approach (Li et al. 2011) and *via* an enzyme purification approach (Kanamasa et al. 2008; van der Straat and de Graaff, personal communications). In the genome of *A. terreus* two putative transporters flank the *cadA* gene. Recently it was shown that especially the putative mitochondrial transporter *mttA* is crucial for an efficient itaconic acid production process in *A. niger* and to minor extend the putative plasmamembrane transporter *mfsA* also has a positive effect on itaconic acid production (Li et al. 2013; van der Straat and de Graaff, personal communications). In the past several research groups tried to increase the flux through the glycolytic pathway since that might greatly enhance the productivity of acid production. The work of Schreferl-Kunar in 1989 already showed that *A. niger* mutants that were selected on the basis of increased citric acid secretion levels have strongly increased hexokinase and 6-phosho-1-fructokinase activity (Schreferl-Kunar et al. 1989). However, overexpression of 6-phosphofructo-1-kinase in a citric acid producing *A. niger* strain did not lead to increased citric acid levels (Ruijter et al. 1997). Nonetheless, cultivation of *A. niger* on high sugar concentrations showed that the control over the glycolytic flux at the level of 6-phosphofructo-1-kinase was absent under these conditions (Peksel et al. 2002). Apparently mutations in the 6-phosphofructo-1-kinase gene led to an enzyme that was less inhibited by citrate. This was confirmed by Mlakar and Legiša (2006) who showed that of the two described forms of *A. niger* 6-phosphofructo-1-kinases (Mesojednik and Legiša, 2005) one form was moderately inhibited by citrate while the other form proved to be completely resistant to citrate inhibition (Mlakar and Legiša 2006). Later on, a specific truncated version of the *pfkA* gene from *A. niger,* resistant to citrate inhibition but still highly active, was obtained (Capuder et al. 2009). The expression of this modified 6-phosphofructo-1-kinase gene in *A. terreus* resulted in increased itaconic acid production levels (Tevž et al. 2010).

In this manuscript, we report the effect of overexpressing the modified 6-phosphofructo-1-kinase in *A. niger* on organic acid production, when combined with the *cadA* gene from *A. terreus* as well as in combination with the itaconic acid biosynthetic cluster *cadA*, *mttA* and *mfsA*.

## Materials and methods

### Strain

The fungal strain used in this study was *A. niger* CAD4 *(cspA, ΔargB, goxC1, prtF28)* which is deposited at DSMZ with accession number DSM 27587.

### Genetic constructs

The modified 6-phosphofructo-1-kinase gene (accession number HG423570) was obtained by PCR using primer LS_modPFKI_for and LS_modPFKI_rev and *A. niger* N593 genomic DNA as a template. The PCR fragment was cloned into a pUC19 derived expression vector under control of a modified *xlnD* promoter and the *xlnD* terminator of *A.niger*. Plasmids pMTT and pMFS contained the synthetic codon-optimized coding sequences (DNA 2.0) of the putative mitochondrial transporter and putative plasmamembrane transporter respectively both under control of a modified *xlnD* promoter (*) and the *xlnD* terminator of *A. niger* (accession numbers HG423568 and HG423569 respectively). For the construction of plasmid pLS001 the [p_xlnD* – MTT – t_xlnD] fragment was obtained by PCR using pMTT as a template and primers LS_p_xlnD_HindIII_for and LS_t_xlnD_XbaI_rev. This fragment was cloned into pMFS, previously digested with *Hin*dIII and *Xba*I. Plasmids were propagated in DH5α *E. coli.* LB medium supplemented with 100 mg/L ampicillin was used for *E. coli* growth.

### Fungal transformation

Protoplasts were generated using Novozyme-234 for the transformation of *A. niger* CAD4 strain. The pPFKmod plasmid was introduced in *A. niger* by co-transformation as described before (Kusters-van Someren et al. 1991), using the pAL69 plasmid containing the *argB* gene as a primary selection marker. The pPFKmod plasmid was also introduced together with the pLS001 plasmid by co-transformation using pAL69. Selective MMS plates (6.0 g/L NaNO_3_, 1.5 g/L KH_2_PO_4_, 0.5 g/L KCl, 0.5 g/L MgSO_4_.7 H_2_O, 1 mL/L Vishniac solution, 325.2 g/L sucrose, 1.2% (w/v) agar, pH 6.0) were used to select for protoplasts that took up the selection marker plasmid and possibly the pPFKmod plasmid and/or the pLS001 plasmid. Colonies were randomly picked from the transformation plates and re-plated on complete medium (Pontecorvo et al. 1953).

### Selection of transformants

Fresh mycelium was disrupted using Fastprep with glass beads and 400 μL DNA extraction buffer (0.1 M Tris HCl pH 8.0, 1.2 M NaCl, 5 mM EDTA). DNA was extracted using phenol-chloroform extraction. The pellet was washed with 70% cold ethanol, air-dried and re-suspended in 50 μL MQ water.

Transformants were selected by PCR using the extracted genomic DNA as a template. The primers LS_pPFK1_for, located in the *pfkA* gene and LS_txlnD_rev located in the *xlnD* terminator, were used to check for the presence of pPFKmod. The presence of the putative mitochondrial transporter *mttA* was confirmed using primers LS_pMTT_for and LS_pMTT_rev whereas the presence of the putative plasmamembrane transporter *mfsA* was confirmed with primers LS_pMFS_for and LS_pMFS_rev (Table 1).

**Table 1:** Sequences of primers used in this study

**Primer name**	**Sequence**
LS_modPFKI_for	GAGAATGCATATGGCTCCCCCCCAAGC
LS_modPFK_rev	GAGAGCGGCCGCATCATAGTGCCGGCACAGACC
LS_p_xlnD_HindIII_for	GAGAAAGCTTCGAATGAGGAGGTGTTGCAG
LS_t_xlnD_XbaI_rev	GAGATCTAGACTGCAGTCGCACTCCCGACC
LS_pPFK1_for	TGACATGTGCGCTATCATTACC
LS_txlnD_rev	TCCCCTAGAGCCATCAACAG
LS_pMTT_for	ATTAAGACCCGCATGCAATC
LS_pMTT_rev	CTTCTCGTAGACGGGGAACA
LS_pMFS_for	ACCTTCACTAGCTGGCGTGT
LS_pMFS_rev	GACATCCGTGGGACTGAACT
LS_q_pfkA_F	CGTGAGAACAAGATCTTGCG
LS_q_pfkA_R	CGCATTCTCTTGTTCTCTGG
LS_q_gotra_F	TTTTCAGTCTGGCTGCTCCT
LS_q_gotra_R	CTGTTTTCCTGCATCGTGTG
LS_q_kan_F	AGCATTACGCTGACTTGACG
LS_q_kan_R	AGGTGGACCAGTTGGTGATT

The transformants containing the genes of interest were re-plated on complete medium. Fungal spores were harvested after 4 days of growth at 30°C.

### Growth experiment

The different *A. niger* transformants and controls were grown, after inoculation of 10^6^ spores per mL, in 1L Erlenmeyer flasks containing 200 mL PM medium (Ruijter et al., 1999) (1.2 g NaNO_3_, 0.5 g KH_2_PO_4_, 0.2 g MgSO_4_.7 H_2_O, 0.5 g Yeast extract and 40 uL Vishniac per liter) (Vishniac and Santer, 1957) with 100 mM sorbitol as a carbon source. The expression of the different recombinant genes studied in this work was induced, 18 hours after inoculation (t=0), with 50 mM xylose. All *A. niger* strains were grown for 5 days at 30°C and 250 rpm. Samples for HPLC analysis were taken at 6 h, 30 h, 54 h and 78 h after induction.

### Metabolite analysis with HPLC

High-pressure liquid chromatography (HPLC) was used to determine the extracellular concentrations of sorbitol, xylose, citric acid, itaconic acid, *cis*-aconitic acid, pyruvic acid, a-ketoglutaric acid, lactic acid, succinic acid, fumaric acid and oxalic acid in the samples. A Thermo Accela equipped with a Shodex KC-811 column was used. Separations were performed by isocratic elution with 0.01 N H_2_SO_4_ at a flow rate of 0.8 mL ● min^-1^. The detection of the different compounds was done using both a refractive index detector (Spectrasystem RI-150, sample frequency 5.00032 Hz) and a UV–VIS detector (Spectrasytem UV1000, λ = 210 nm). Crotonate (6 mM) was used as an internal standard.

### RNA extraction and cDNA preparation

Frozen mycelium was placed in 2 mL tubes prefilled with 1 mm silica spheres (Lysing Matrix C, MP) and homogenized in 1 mL of peqGOLD TriFast DNA/RNA/protein purification system reagent. A FastPrep-24 Instrument (MP) was used to distupt the mycelium. RNA was isolated according to the manufacturer’s instructions. The extracted total RNA is spiked with a synthetic control RNA transcript, a bacterial kanamycin synthetase-encoding gene fused to a eukaryotic poly (A) tail (Promega), to correct for various efficiencies of reverse transcription or PCR itself (Huggett et al. 2005). cDNA was synthesized using the RevertAid H Minus first-strand cDNA synthesis kit (Thermo Scientific) according to the manufacturer’s instructions.

### qPCR analysis

Transcript levels were determined in triplicate for the gene of interest *pfkA* gene as well as for a reference gene (GOTRA) and the synthetic control RNA transcript using the Rotor-Gene Q Cycler. The qPCR mixture contained 7.5 μL 2x Absolute QPCR SYBR Green mix (Thermo Scientific), 100 nM forward and reverse primers and 2.5 μL 100 times diluted cDNA. Primers LS_q_pfkA_F and LS_q_pfkA_R were used to determine the transcript level of the *pfkA* gene and primers LS_q_gotra_F and LS_q_gotra_F were used to determine the transcript level of the reference gene An02g04120 (van der Veen et al. 2009). The transcript levels of the synthetic kanamycin gene were determined using primers LS_q_kan_F and LS_q_kan_R (Table 1).

Water and SDS samples were used as controls. The qPCR cycling program was as follows; 15 min initial polymerase activation at 95°C followed by 40 cycles of 95°C for 15 sec, 58°C for 15 s and 72°C for 20 s. The calculations were done using the method of Pfaffl (Pfaffl, 2001) using the expression level of *pfkA* in the parent strain CAD as the reference.

## Results

### Transformants harboring *cadA* and the modified *pfkA* gene

**Figure 1:**
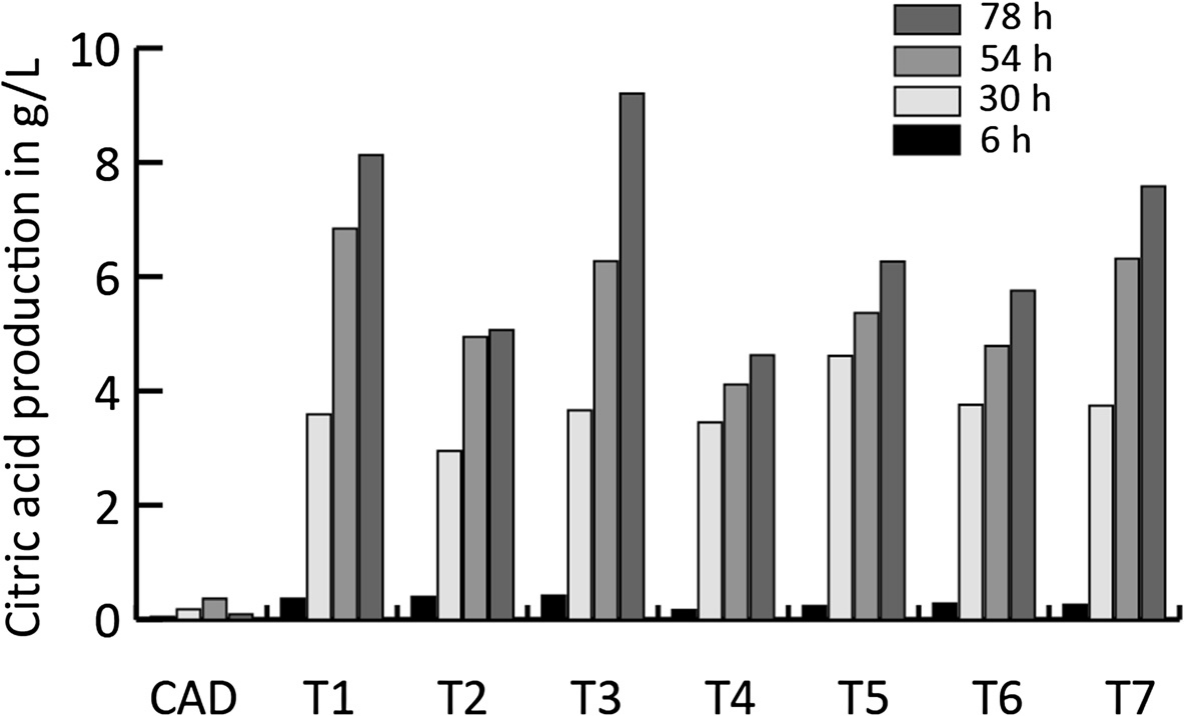
**Citric acid production in g/L of the transformants harboring *cadA *** **and the modified *pfkA *** **followed in time (6, 30, 54, 78 hours after induction).**

**Figure 2:**
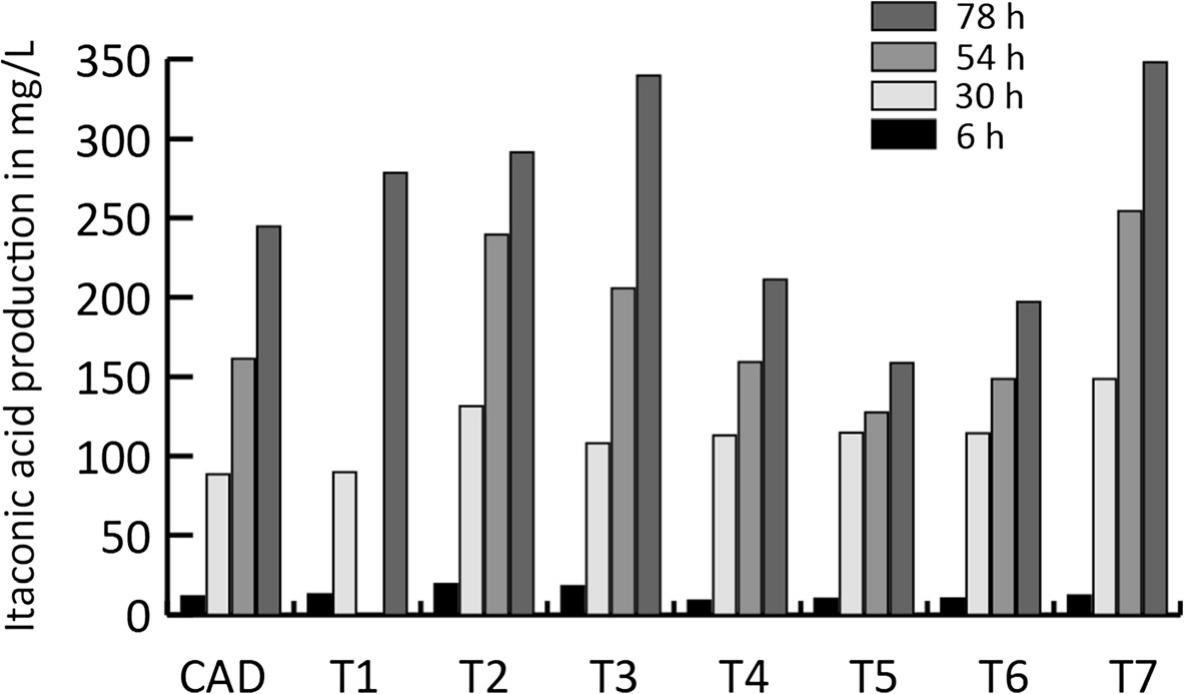
**Itaconic acid production in mg/L of the transformants harboring *cadA *** **and the modified *pfkA *** **followed in time (6, 30, 54, 78 hours after induction).**

**Figure 3:**
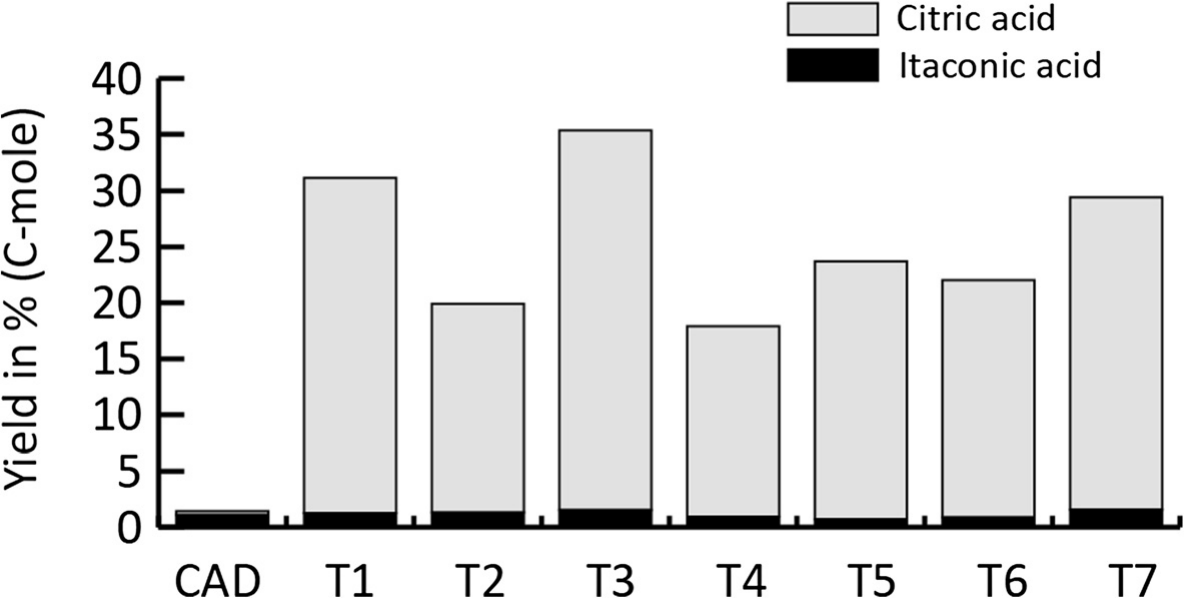
**Calculated C-mole yield (%) of itaconic acid and citric acid for each transformant harboring the modified *pfkA *** **gene in combination with *cadA *** **compared to the control strains that only contain the *cadA *** **gene at t=78.**

Extracellular itaconic acid and citric acid levels were monitored during a time course experiment that included seven *A. niger* CAD4 transformants (named T1 to T7), carrying both the *cadA* gene from *A. terreus* and the modified *pfkA* gene from *A. niger,* plus the parent strain as a control. Overexpression of the modified *pfkA* gene in the *A. niger cadA* harboring strain led to a strongly increased citric acid production when compared to the parent strain CAD (Figure 1). All transformants produced significantly higher levels of citric acid. The levels of citric acid produced range between the 4.6 and 9.2 g/L while the parent strain produced only 0.1 g/L under these conditions. However, in contrast to the high increase in observed citric acid production, the combination of the modified *pfkA* gene and the *cadA* gene from *A. terreus* did not lead to an increase in itaconic acid levels, as shown in Figure 2 Four of the analyzed transformants produce slightly more itaconic acid as compared to the parental strain, while three of the transformants produced less itaconic acid 78 hours after induction. The parental strain reached extracellular itaconic acid levels of 240 mg/L while the transformants produced between 159 and 348 mg/L. The combined C-mol yield of citric acid and itaconic acid is strongly improved as a result of the introduction of the modified *pfkA* gene (Figure 3). The best performing transformant reached a yield of 35%.

### Transformants expressing the itaconic acid biosynthetic cluster *cadA, mttA, mfsA* in combination with the modified *pfkA* gene

**Figure 4:**
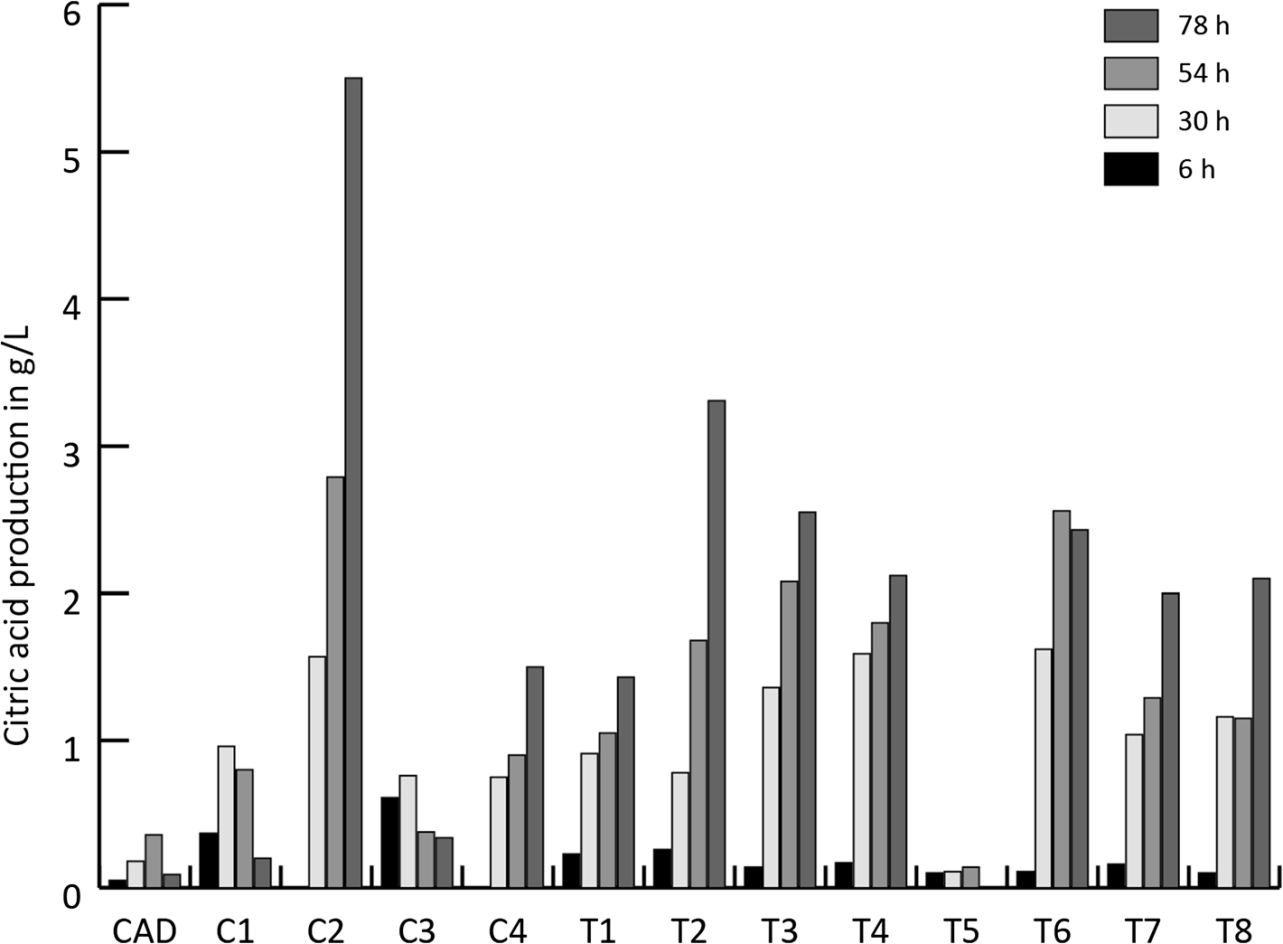
**Citric acid production in g/L of the transformants harboring the complete itaconic acid biosynthesis cluster *cadA, mttA and mfsA *** **respectively and the modified *pfkA *** **(T1-T8) compared to the transformants containing only *cadA, mttA *** **and *mfsA *** **genes (C1, C2, C3, C4) followed in time (6, 30, 54, 78 hours after induction).**

**Figure 5:**
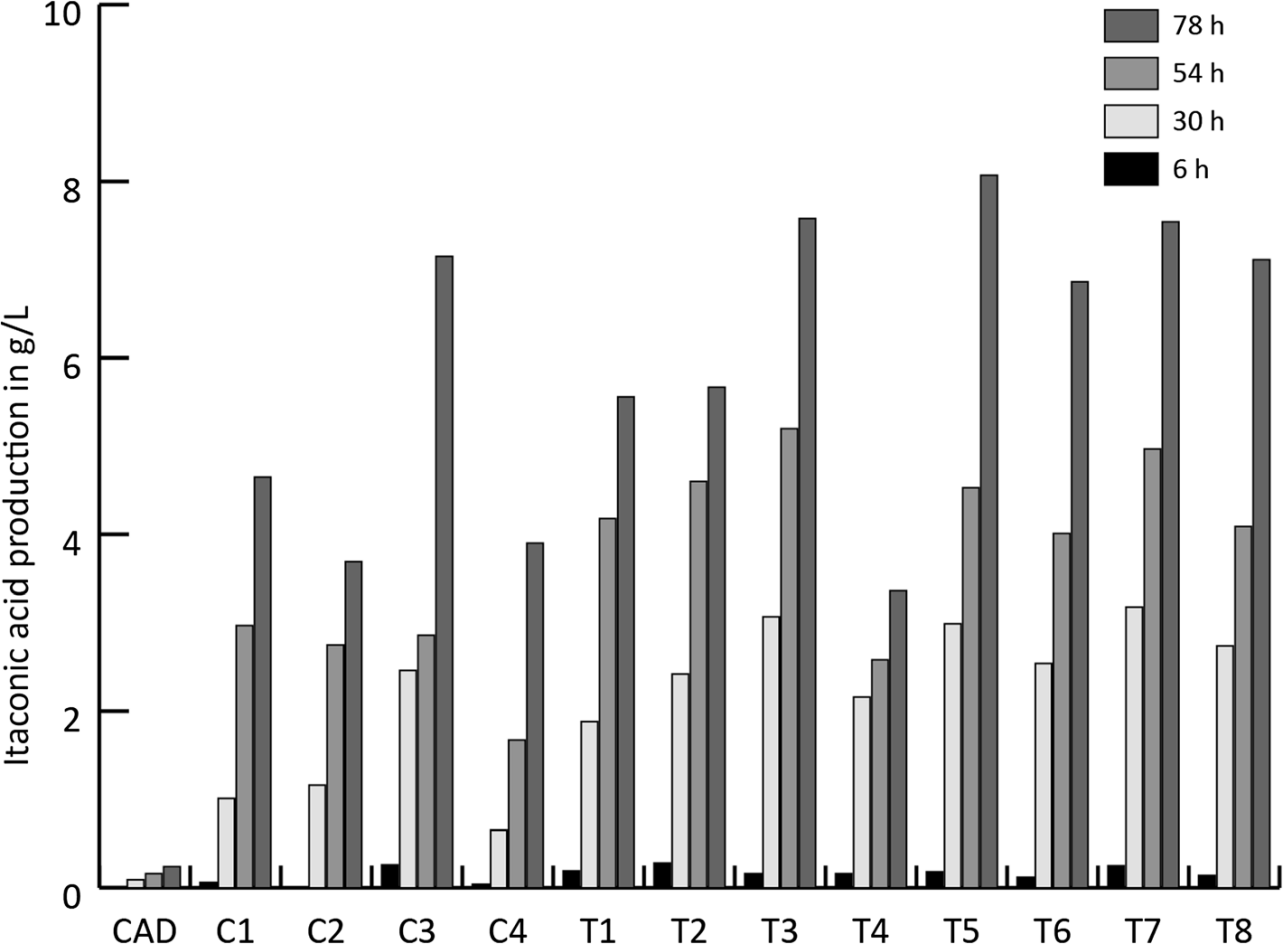
**Itaconic acid production in g/L of the transformants harboring the complete itaconic acid biosynthesis cluster *cadA, mttA and mfsA *** **respectively and the modified *pfkA *** **(T1-T8) compared to the transformants containing only *cadA, mttA *** **and *mfsA *** **genes (C1, C2, C3, C4) followed in time (6, 30, 54, 78 hours after induction).**

**Figure 6:**
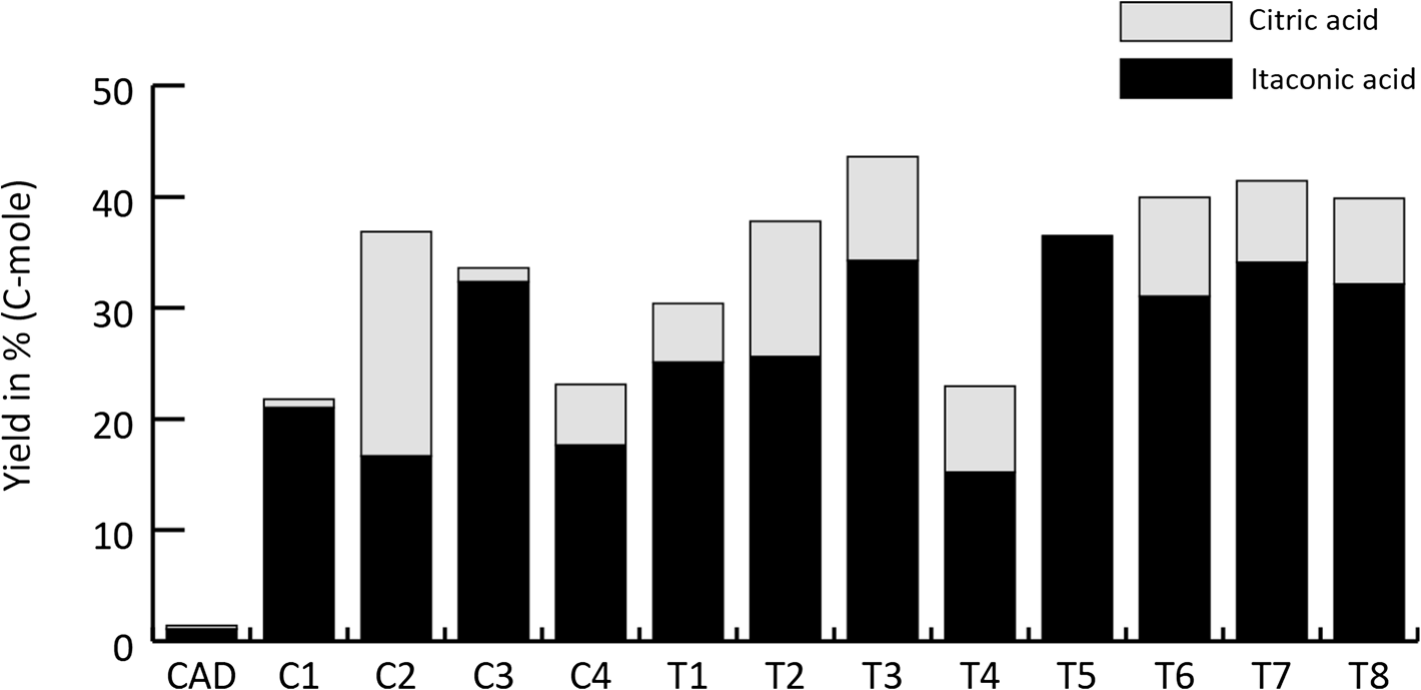
**Calculated C-mol yield (%) of itaconic acid and citric acid for each transformant harboring the complete itaconic acid biosynthesis cluster *cadA, mttA *** **and *mfsA *** **respectively and the modified *pfkA *** **(T1-T8) compared to the control strains that only contain the *cadA, mttA *** **and *mfsA *** **genes (C1, C2, C3, C4) at t=78.**

Both the strains harboring the itaconic acid production cluster *cadA, mttA* and *mfsA* (C series) as well as the transformants carrying the modified *pfkA* gene together with the itaconic acid biosynthetic cluster (T series) have very variable citric acid production levels (Figure 4). The largest difference was found in the strains that also express the itaconic acid biosynthetic cluster, in which the citric acid level varies between 0.2 and 5.5 g/L at 78 hours after induction. The strains that also express the modified *pfkA* in combination with the itaconic acid biosynthetic cluster produce significantly higher amounts of itaconic acid (Figure 5) at sampling time point 30 h and 54 h after induction compared to the strains that only harbor the itaconic acid biosynthetic cluster (T-test, p = 0.04 for 30 h and p = 0.003 for 54 h). At t=78 h after induction there were no significantly increased levels of itaconic acid observed (p = 0.15). The total yield, expressed in C-mol, of itaconic acid and citric acid in the cluster strains (C series) ranged between 22% and 37% while the yield of the cluster plus the modified *pfkA* transformants (T series) ranged between 23% and 44% at t=78 h. This was statistically not significantly higher (p = 0.14) (Figure 6). However, the total C-mol yield of itaconic acid and citric acid is only significantly higher for the *pfkA* transformants at t=30 h and t=54 h (p = 0.03 and p = 0.03 respectively, data not shown). In addition, the productivity levels were significantly higher (p = 0.03) for the transformants expressing the itaconic acid biosynthetic cluster and the modified *pfkA* genes compared to the strains that lack the modified *pfkA* gene.

**Figure 7:**
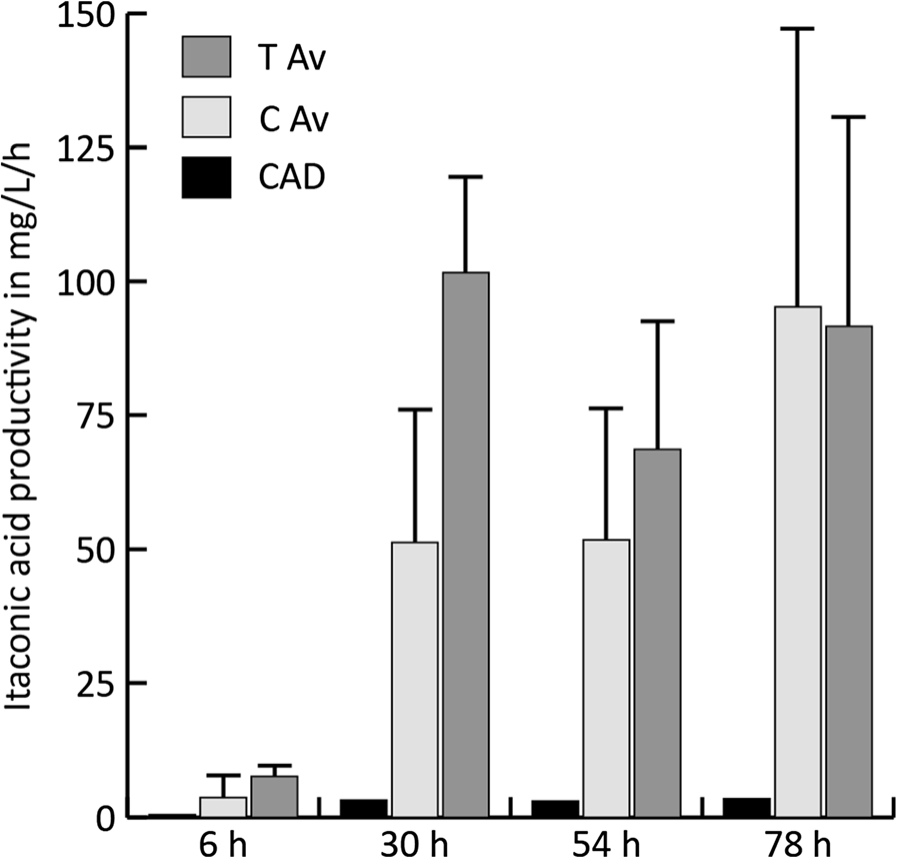
**Average itaconic acid productivity levels in mg/L/h for the transformants harboring the cluster genes *cadA, mttA, mfsA *and the modified *pfkA *(T Av) compared to the average productivity of the transformants harboring only the cluster genes *cadA, mttA, mfsA *(C Av).** At time point t=30 h the difference in productivity is significant (T-Test, p=0.03).

**Figure 8:**
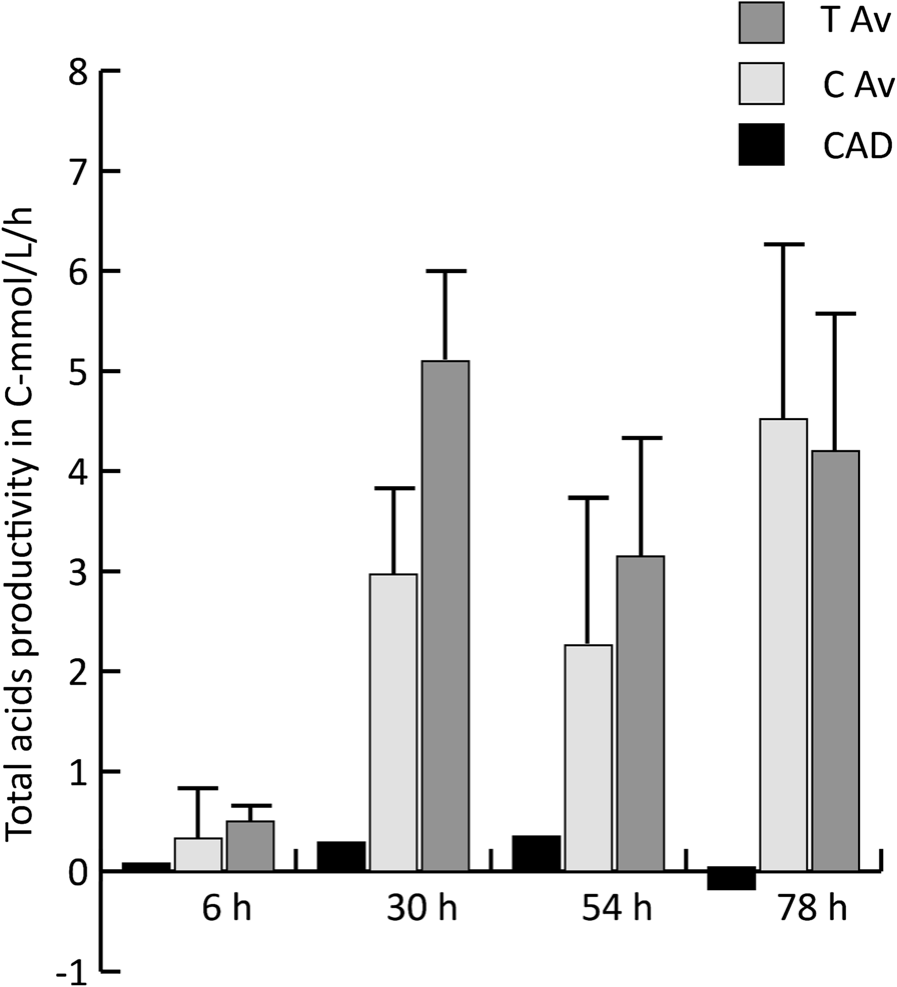
**The average total acid production in C-mmol/L/h for the transformants harboring the cluster genes *cadA*, *mttA*, *mfsA *and the modified *pfkA *(T Av) compared to the average productivity of the transformants harboring only the cluster genes *cadA*, *mttA*, *mfsA *(C Av).** At time points t=30h the difference in productivity is significantly different (T-Test, p=0.01).

The highest itaconic acid productivity level within the modified *pfkA* recombinant strains (T series) was reached 78 hours after induction by one single transformant (147 mg/L/h), while, on average, the highest productivity levels for this group of strains was already reached after 30 hours (102 mg/L/h) (Figure 7). The total organic acid productivity, expressed in C-mmol/L/h (Figure 8), increased significantly at t=30 h (p = 0.01) for the transformants (T series) compared to the strains harboring only the cluster genes (C series). The maximum total organic acid productivity at t=30 h was 6.25 C-mmol/L/h and corresponded to one of the transformants harboring the cluster genes and the modified *pfkA* gene, while the best producing strain, expressing only itaconic acid biosynthetic cluster, achieved a maximum of 3.9 C-mmol/L/h.

**Figure 9:**
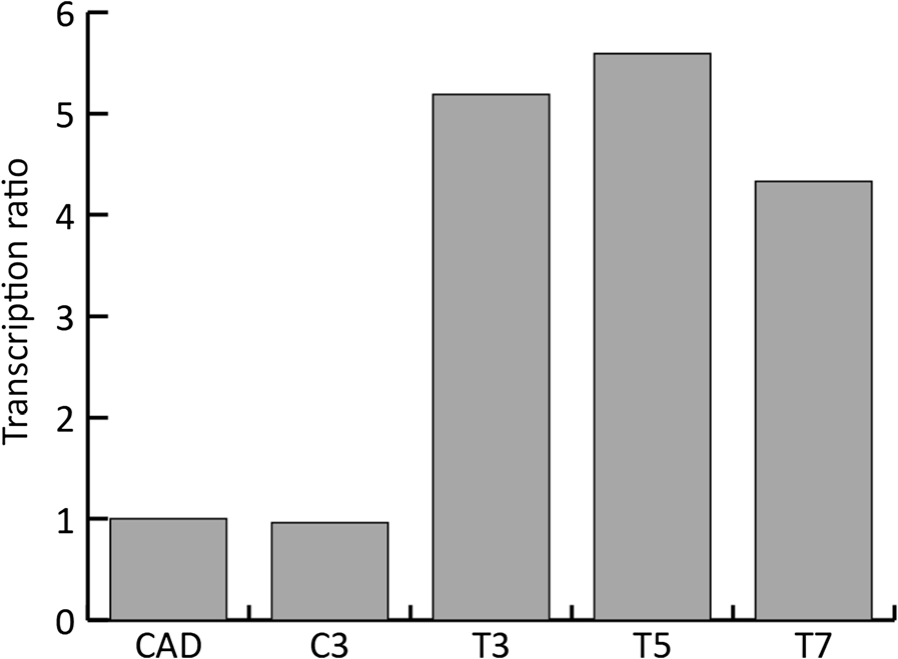
**Relative transcription levels of the *pfkA *gene in the different transformants compared to the parent strain CAD that was used as the reference strain.** Samples were taken 30 hours after induction, which was the time point where the highest itaconic acid productivity was observed. C3 is the strain that contains the cluster genes *cadA*, *mttA* and *mfsA*. The transformants T3, T5 and T7 are the strains that express the cluster genes *cadA*, *mttA* and *mfsA* as well as the modified *pfkA*.

The transcription ratios of the *pfkA* gene of the three transformants that gave the highest itaconic acid productivity at time point 30 h (T3, T5 and T7) were compared with the highest itaconic acid producing strain that expresses the cluster genes *cadA*, *mttA* and *mfsA* (C3) and with the parent strain CAD. The results showed that there is no difference in *pfkA* transcription between the CAD strain and the C3 strain. The transformants T3, T5 and T7 gave an increased transcription level of a factor 5.2, 5.6 and 4.3 respectively, compared to the parent strain CAD (Figure 9).

## Discussion

Our results show transformants that overexpress the modified *pfkA* gene in combination with *cadA* have significantly increased citrate secretion levels. This confirms that the modified *pfkA* gene has a similar effect on citric acid production in our strain compared to what was found before by Capuder et al. (2009). They were able to show that the overexpression of the modified *pfkA* gene in the *A. niger* 158 strain resulted in a 70% increase in citrate secreted into the fermentation broth after 300 hours of growth.

In our research the effect was far more evident but since the experimental set-up was quite different it is difficult to compare both results. The overexpression of the modified *pfkA* in the CAD strain also had a strong effect on the citrate yield: levels up to 35% were achieved while the CAD strain only reached a yield of about 2%. As shown before, in particular the putative mitochondrial transporter *mttA* and to a lesser extent also the putative plasmamembrane transporter *mfsA* play a crucial role in itaconic acid production (Li et al. 2013; van der Straat and de Graaff, personal communications). This was confirmed by the results reported in this work, since itaconic acid production in the strains harboring the modified *pfkA* gene and the *cadA* gene from *A. terreus* was not improved when compared to the parent strain that only harbored the *cadA* gene. Since both transporters are crucial for itaconic acid production, the modified *pfkA* gene was also co-expressed with the complete itaconic acid biosynthetic cluster of *A. terreus* consisting of *cadA*, *mttA* and *mfsA*. The effect of these metabolic modifications on citrate production varied. This can be expected because the construct carrying the genes is randomly integrated in the genome of *A. niger* and its copy number varies.

However, we also do find an effect of these metabolic modifications on itaconic acid production. The transformants series harboring the modified *pfkA, cadA, mttA* and *mfsA* produced significantly more itaconic acid than the strains harboring *cadA, mttA* and *mfsA* both at 30 and 54 hours after induction. However, 78 hours after induction the itaconic acid production of the T series and the C series strains was not significantly different. This result shows that the modified 6-phosphofructo-1-kinase has a positive effect on itaconic acid production only in the early stages of production. The average highest itaconic acid productivity reached by the T series transformants occurred also at early time points. In particular, at 30 hours after induction, a significant difference in productivity, caused by the overexpression of the modified *pfkA* gene, was observed.

The transcription levels were determined for the *pfkA* gene using a set of primers that bind both to the cDNA of the endogenous *pfkA* gene as well as to the cDNA of the modified *pfkA* gene. This assumes that the transcription levels of the endogenous *pfkA* gene do not change after transformation. The transcription ratio (1.0) of the strain harboring the cluster genes *cadA*, *mttA* and *mfsA* compared to the parent strain *cadA* (1.0) showed that the expression of the transporters do not influence the transcription level of *pfkA*.

A positive effect on the itaconic acid productivity was also observed when the modified *pfkA* gene from *A. niger* was expressed in *Aspergillus terreus* (Tevž et al. 2010). As suggested in the report of the US Department of Energy (Werpy and Petersen 2004) an itaconic acid productivity of 2.5 g/L/h should be achieved before the process is economically feasible. Under the conditions tested in the present study the productivity was only a factor 17 lower than the desired productivity. Since our growth conditions are merely designed to test the effects of the metabolic modifications at lab scale, several improvements, such as the use of more optimal culture conditions and *A. niger* genetic backgrounds, could be done in order to increase the itaconic acid production to the desired industrial levels. In this study growth media contains 100 mM sorbitol and 50 mM xylose as a carbon source and inducer, while glucose based media are predominantly used for the production of citric acid and itaconic acid. Using different carbon sources in higher concentrations might further improve the itaconic acid productivity. The strain we used is not an industrial citrate-producing strain and the growth experiment in this study was done in shake flasks. In summary, the usage of an industrial citrate-producing *A. niger* strain in an optimized and controlled fermentation process should allow itaconic acid productivity levels of 2.5 g/L/h and thus create an economically feasible production process.

## Competing interests

The authors declare that they have no competing interests.

## Authors’ contributions

LS, JT, TS designed and performed the experimental work and participated in writing the manuscript. LG designed the study and participated in writing of the manuscript. All authors read and approved the submission of the manuscript.
